# Exogenously Applied GA_3_ Enhances Morphological Parameters of Tolerant and Sensitive *Cyclamen persicum* Genotypes under Ambient Temperature and Heat Stress Conditions

**DOI:** 10.3390/plants11141868

**Published:** 2022-07-18

**Authors:** Mihaiela Cornea-Cipcigan, Mirela Irina Cordea, Rodica Mărgăoan, Doru Pamfil

**Affiliations:** 1 Department of Horticulture and Landscaping, Faculty of Horticulture, University of Agricultural Sciences and Veterinary Medicine, 400372 Cluj-Napoca, Romania; mihaiela.cornea@usamvcluj.ro; 2 Laboratory of Cell Analysis and Spectrometry, Advanced Horticultural Research Institute of Transylvania, University of Agricultural Sciences and Veterinary Medicine of Cluj-Napoca, 400372 Cluj-Napoca, Romania; 3 Research Centre for Biotechnology in Agriculture Affiliated to Romanian Academy, University of Agricultural Sciences and Veterinary Medicine, 400372 Cluj-Napoca, Romania; presedinte.arfc@academia-cj.ro

**Keywords:** crop tolerance, germination, heat mapping, heat stress, plant development

## Abstract

*Cyclamen* genus is part of the Primulaceae family consisting of 24 species widely cultivated as ornamental and medicinal plants. They also possess high plasticity in terms of adaptability to alternating environmental conditions. In this regard, the present study investigates the germination and morphological parameters of heat-tolerant and heat-sensitive *Cyclamen persicum* accessions in the presence of different GA_3_ solutions (0, 30, 70 and 90 mg/L) under ambient temperature and heat stress conditions. Heat-tolerant genotypes, mainly C3-Smartiz Victoria (6.42%), C15-Merengue magenta (6.47%) and C16-Metis silverleaf (5.12%) had the highest germination rate with 90 mg/L GA_3_ treatment compared with control. Regarding heat-sensitive genotypes, C11-Verano (5.11%) and C13-Metis Origami (4.28%) had the lowest values in mean germination time, along with the Petticoat genotypes C1 (73.3%) and C2 (80.0%) with a high germination percentage. Heat-tolerant genotypes positively responded to GA_3_ (70 and 90 mg/L) even under heat stress conditions, by their higher values in plant height, an ascending trend also seen in heat-sensitive genotypes under GA_3_ treatment (70 and 90 mg/L). According to the hierarchical clustering, several heat-tolerant genotypes showed peculiar behavior under heat stress conditions, namely C3 (Smartiz Victoria), C7 (Halios falbala) and C8 (Latinia pipoca) which proved to be susceptible to heat stress even under GA_3_ application, compared with the other genotypes which showed tolerance to higher temperatures. In the case of heat-sensitive genotypes, C4 (Smartiz violet fonce), C6 (Metis blank pur), C11 (Verano) and C13 (Metis origami) possessed higher positive or negative values compared with the other heat-sensitive genotypes with increased doses of GA_3_. These genotypes were shown to be less affected by heat stress, suggesting their positive response to hormone treatment. In conclusion, the above-mentioned genotypes, particularly heat-tolerant C15 and heat-sensitive C2 with the highest germination capacity and development can be selected as heat-resistant genotypes to be deposited in gene banks and used in further amelioration programs under biotic and/or abiotic stresses to develop resistant genotypes.

## 1. Introduction

Abiotic stress has become a key area of concern in crop production as a result of global warming. Substantial research is being carried out to develop approaches that deal with abiotic stresses, to develop heat-, sun-, and drought-tolerant cultivars, adjust crop calendars, and resource management approaches, among other things [1]. As these practices are costly and time-consuming, recent studies denote that the introduction of high-yield phenotyping techniques, along with other non-destructive methods, has increasingly taken over the plant phenomics area, where novel technologies such as non-invasive imaging, spectroscopy and high computation performance are combined to assess phenotypic performances at high resolution, flow and accuracy. This will aid breeders and plant scientists to conduct phenotypic experiments with large plant populations in different environments to non-destructively monitor the performance of plants over time [2].

*Cyclamen* is widely distributed in the Mediterranean regions of Cyprus, Rhodes and (Eastern) Crete, as well as coastal areas of the Eastern Aegean and from southern Turkey to northern Israel [3]. It presents adaptation to habitat modifications and environmental changes [4], but its exploitation has had negative effects on native populations. Special attention is also regarded to their antioxidant and strong anticancer activity [5,6,7,8], but as they mostly germinate from seeds and have a slow development, they are hard to use extensively. Moreover, being sensitive to inbreeding depression, germination capacity of pollen grains is employed for efficient artificial hybridization [9]. Although it is highly acclimated to the Mediterranean environment, an increase in temperature, alongside water shortage and other biotic and abiotic factors, might affect both the quality and quantity of the crop, traits that are affected by both temperature and water accessibility [10]. The Schoneveld Breeding Company recently evaluated the effect of high levels of light exposure on leaf temperature in *Cyclamen*. They demonstrated that on sunny days if the light intensity is high, the leaves’ temperature will gradually rise above the air temperature. In this case, the plant cannot maintain enough evaporation to cool and will therefore close the stomata to prevent wilting. This results in an inactive plant for up to 5 h/day due to no water/nutrients uptake because of closed stomata. The optimal temperature in the first period of growth is around 18–20 °C and between 15–20 °C in the flowering period. Therefore, to promote cooler temperatures, up to 50% shading is applied in the summer months, together with lateral and/or roof ventilation [11]. Recent studies demonstrated the correlation between GA application and light exposure to cardinal temperatures and thermal times required for seed germination [4,12].

Molecules that protect plants against several adverse climate conditions are starting to gain interest among plant researchers. Exogenous application of growth regulators like gibberellic acids (GAs) have been demonstrated to be effective in ameliorating abiotic stresses, including in heat-stress induced plants [13,14]. Moreover, gibberellins are key plant regulators that control the developmental stage of plants [4]. They have been extensively used in economically important crops and ornamentals due to their positive effects in early seed germination [15,16], reduced juvenile stage [17], leaf and root development and extension [16,18], early flowering and fruit formation [19,20]. It has been shown that mutant plants deficient in GA have a diminutive phenotype and blossom late. A growing demand for patterned foliage and prolonged vase life in *Cyclamen* cut-flowers has led researchers to design new amelioration strategies to meet market demands [21]. GA_3_ foliar applications increased flower stem length in cut flowers cultivated in open-fields and delayed leaf browning and senescence [22].

Seed-priming is one of the most low-cost and low-risk methods of improving germination, seedling development and yield [23]. Exogenously applied GA_3_ enhances seed germination and dormancy release, thus highlighting the importance, fast effectiveness and low cost of seed-priming in multiple crops [24]. The long-term effect of seed priming with GA_3_ was assessed in multiple crops to determine the plant growth and production [15], which has been demonstrated to improve germination and growth parameters of shoot and root length, and seedling fresh and dry weights [15,25]. Furthermore, seed priming has been shown to enhance resistance to abiotic stress via various pathways involved in different metabolic processes, with best results in early and uniform germination [26]. Thus, seed simulation modeling, a topic of interest in quantifying germination and growth dynamics of plants, may assist researchers to forecast seed germination and final germination potential at the end of the experiment. Thus, it is critical to assess the genetic diversity of planted germplasm for heat-stress resistance and to choose genotypes with higher levels of heat tolerance. In this regard, the treatment estimate provides a framework for evaluating the genetic potential of germination and quality-related plant characteristics under optimum and heat-stress circumstances. The aims of the study were: to non-destructively evaluate the germination capacity (germination capacity and mean germination time) and plant development (seedling vigor index, leaf area, plant, petiole and root lengths) of heat-tolerant and heat-sensitive *Cyclamen* genotypes subjected to ambient temperature (AT) or heat stress (HS) conditions; to select the most advantageous GA_3_ dosage applied; to evaluate the cultivated germplasm’s genetic diversity for stress tolerance; and to select genotypes which possess increased tolerance to heat.

## 2. Results

### 2.1. Variation in Germination Parameters of Heat-Resistant and Heat-Sensitive Cyclamen Genotypes under AT and HS

Heat-resistant and heat-sensitive *Cyclamen* responded variably to HS conditions and GA_3_ treatments (Table 1). Under HS heat-resistant genotypes responded positively to GA_3_ treatment concentrations in a dose-dependent and specie-specific manner. Thus, under control, relatively low germination percentages were noticed especially in C10 (53.4%) and C14 (60%), and the highest were found in both C8 and C15 with 86.7%. Following treatment with 30 mg/L GA_3_, the highest germination was observed in C10 (93.3%) and the lowest in C7 (60.0%). Increased GA_3_ concentrations (70 and 90 mg/L) had a positive effect in all genotypes except C3 which presented a slightly lower germination between 80 and 83.3%. Regarding the heat-sensitive genotypes, significant differences were noticed between temperature conditions and hormone treatment. Under HS, the heat-sensitive genotypes presented lower germination percentages even among those subjected to GA_3_ treatments, except C1, C2 and C4. The highest germination and resistance to HT was noticed in C2 in all GA_3_ concentrations, followed by C4 which presented the highest germination with 30 and 70 mg/L GA_3_, whereas C1 had the best germination performance under 70 and 90 mg/L GA_3_ concentrations with 73.3 and 80.0% germination percentages, respectively. In heat-resistant genotypes under AT, the greatest germination percentage was observed under 70 mg/L GA_3_ in C3, C7, C10 and C16. Conversely, relatively low germination was noticed in C14 and C15 under all treatment concentrations. Treatment with 30 mg/L GA_3_ concentrations revealed relatively close values with control in all genotypes. Compared with the other concentrations, the highest 90 mg/L GA_3_ concentration presented the best results in all genotypes with the highest germination percentage in C9. Under AT, the highest germination percentage was noticed using 30 and 70 mg/L GA_3_ with best results in C4, C6 and C13. Conversely, under the 90 mg/L GA_3_, the highest germination was noticed in C2 and C11.

The period required for germination under HS mainly corresponded to the germination percentage (Table 1). Thus, genotypes under control presented a delayed germination as noticed by the increased mean germination time (MGT) values (8.42–19.39%). Following GA_3_ treatment with 30 mg/L, a lower germination rate was noticed in C9 and C14 genotypes with an MGT of 26.24% and 16.77%, respectively. Conversely, genotypes C3, C7 and C16 presented lower MGT values of 7.50%, 6.13% and 6.47%, respectively, corresponding to an earlier germination rate. Treatment with 70 mg/L GA_3_ presented a higher germination rate as seen by the lower values in MGT, except C10 and C14 which had a lower germination rate compared with control and the other treatments. As seen in the previous GA_3_ treatments, genotypes C3, C15 and C16 had the earliest germination with 90 mg/L GA_3_ compared with control and the other genotypes subjected to different hormone concentration treatments. In heat-resistant genotypes, the lowest MGT values (3.03–9.89%) were noticed under 70 mg/L GA_3_ which presented an earlier germination compared with control and the other treatments. The lowest MGT values correspond to the highest germination percentages (i.e., shorter period required for germination) compared with control which presented higher MGT values in most genotypes, except in C7 and C16. Under 30 mg/L GA_3_ treatment, all genotypes presented earlier germination, except C14 which showed a higher MGT (9.89%). Lastly, C14–C16 genotypes showed earlier germination rates under the highest GA_3_ level (90 mg/L). In heat-resistant genotypes, under HS the highest MGT values (i.e., delayed germination) were recorded under control as also seen by the lowest germination percentages, except C7 which had a slightly lower MGT of 8.42%. Treatment with 30 and 70 mg/L GA_3_ showed significantly higher MGT values in almost all genotypes, except C1 and C12 which at both concentrations had the lowest MGT values compared with control presenting an increased germination rate. Finally, using 90 mg/L GA_3_, the lowest MGT values were noticed in C11 (Verano) and C13 (Metis Origami) making them favorable to be used in future amelioration programs. Significant differences were noticed in the heat-sensitive genotypes which had the highest germination rate under AT as shown by the relatively low MGT, especially in the case of 70 mg/L GA_3_. Under control, the lowest MGTs were noticed in C2, C6 and C12 with values between 3.86 and 4.77 which also had a relatively high germination percentage (80.0–93.3%). Under treatment with 30 mg/L GA_3_, the genotypes had a slightly higher germination rate (lower MGT) compared with control, except C5 which had a delayed germination but a higher germination percentage. As seen by the higher germination percentage values (93.3–100%), the heat-sensitive genotypes had the earliest germination rate under the 70 mg/L GA_3_ treatment (2.01–9.89) compared with control and other treatments. The final GA_3_ presented the lowest MGT values in C14 and C16. Overall, genotypes C3 (Smartiz Victoria), C15 (Merengue magenta) and C16 (Metis silverleaf) had the highest germination rates compared with control and GA_3_ treatments, which makes them suitable to be selected as heat-resistant genotypes and used further in several amelioration programs under biotic and/or abiotic stresses, especially drought stress to develop resistant genotypes. Overall, heat-sensitive C11 and C13, which had the lowest MGT values, along with C1 and C2 with a high germination percentage can be selected as genotypes which might be used in future studies to assess their resistance under heat and/or drought stress, but also subjected to different growth hormone regulators. Finally, all heat-resistant genotypes presented high germination rate and percentages making them economically important and suitable to be cultivated in arid regions with elevated temperatures.

### 2.2. Variation in Plant Development of Heat-Resistant and Heat-Sensitive Cyclamen Genotypes under AT and HS

**Figure 1:**
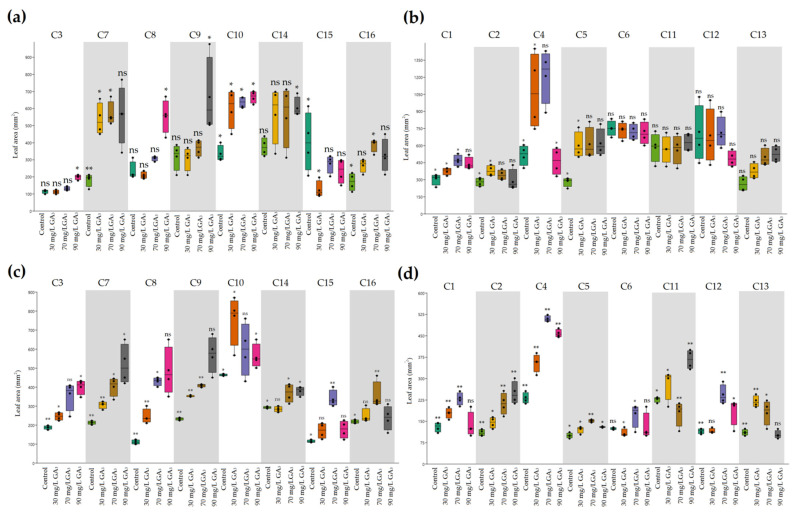
The effects of ambient temperature and high temperature stress and exogenously applied GA_3_ on leaf area of heat-tolerant (**a**,**c**) and heat-sensitive (**b**,**d**) genotypes. * Significant at 5% probability level; ** significant at 1% probability level; ns, no significant difference.

The plants’ height was measured from the beginning of the tuber to the highest point of the leaf (Table 2). The development of heat-resistant and heat-sensitive genotypes was significantly influenced by GA_3_ treatment concentrations. Under HS, significantly lower values were noticed in the heat-resistant genotypes under control, except C14 which had an increased height (6.27 cm) compared with the other genotypes. Under 70 mg/L GA_3,_ the genotypes that presented the highest development were C9, C10 and C14 with values between 12.79 and 15.43 cm. The last treatment significantly increased the plants’ height, except genotypes C3 and C7 with values between 5.41 and 7.01 cm. Under HS, significantly lower values were observed in heat-sensitive genotypes under control, which persisted even in those subjected to 30 mg/L GA_3_ treatment. As it can be foreseen, in the subsequent treatments of 70 and 90 mg/L GA_3_, the plants developed in a dose-dependent manner. The most sensitive to heat were C1, C2 and C12 which presented relatively lower development compared with the other genotypes. Under AT, the heat-resistant genotypes under control presented significantly lower development compared with genotypes subjected to hormone treatment. Under 30 mg/L GA_3,_ the C8, C10 and C14 positively responded to hormone treatment with values between 11.15 cm and 13.25 cm compared with the other genotypes which had relatively close values with control. The subsequent treatment concentrations (70 and 90 mg/L GA_3_) had a positive influence in all tested genotypes in a dose-dependent manner. In the case of heat-sensitive genotypes, a significantly lower development was noticed in control plants under AT. Similar development was also noticed with 30 mg/L GA_3_, with increased plant height noticed solely in C2. The ensuing 70 mg/L had a positive influence regarding the plant’s height in all genotypes with regard to C4 (10.08 cm). The same case was noticed with increased hormone treatment of 90 mg/L in all genotypes. Overall, in the case of heat-resistant genotypes, the plants had the best development under the 90 mg/L GA_3_, as also seen by the high levels in leaf area (Figure 1), except C3 and C7 which might be resistant to moderate heat.

Leaf area was significantly influenced by GA_3_ treatment concentrations in heat-resistant genotypes in a dose-dependent manner, except C14 for which treatment at 90 mg/L reduced the leaf area (Figure 1c). Regarding the heat-sensitive genotypes, significantly lower leaf areas were noticed compared with heat-resistant genotypes. Although the leaf area increased with GA_3_ concentrations, reduced levels were noticed under 90 mg/L in almost all genotypes, except C1 (Figure 1d).

**Figure 2:**
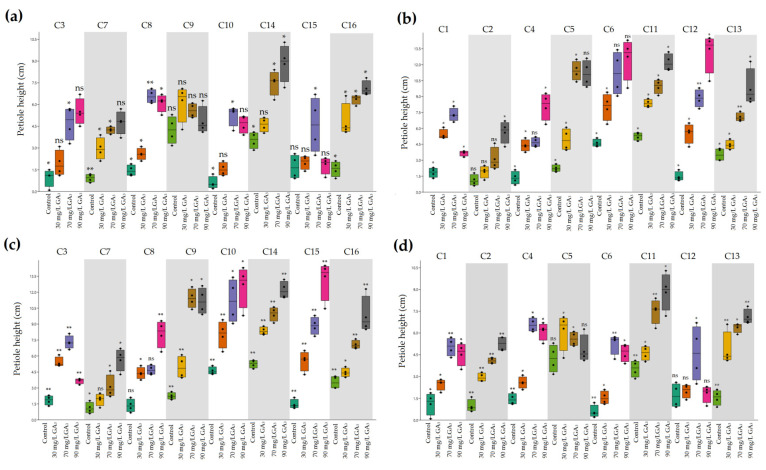
The effects of ambient temperature and high temperature stress and exogenously applied GA_3_ on petiole height of heat-tolerant (**a**,**c**) and heat-sensitive (**b**,**d**) genotypes. * Significant at 5% probability level; ** significant at 1% probability level; ns, no significant difference.

Under HS, increased values of petiole length were noticed in heat-resistant genotypes with increased concentrations of GA_3_, with the highest values under 90 mg/L hormone treatment (Figure 2c). Conversely, heat-sensitive genotypes exhibited relatively low levels in petiole height compared with heat-resistant genotypes. As also seen in heat-resistant genotypes, although higher values were noticed with increased application of GA_3_, reduced levels were recorded at 90 mg/L treatment (Figure 2d).

**Figure 3:**
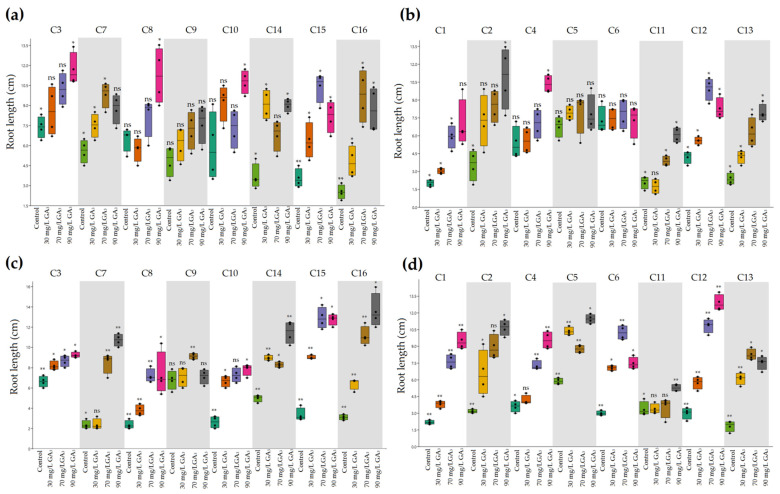
The effects of ambient temperature and high temperature stress and exogenously applied GA_3_ on root length of heat-tolerant (**a**,**c**) and heat-sensitive (**b**,**d**) genotypes. * Significant at 5% probability level; ** significant at 1% probability level; ns, no significant difference.

Regarding root length, significant differences were noticed in heat-resistant genotypes which exhibited longer roots with increased GA_3_ application, except C9 with higher values using 70 mg/L of growth regulator (Figure 3c). In heat-sensitive genotypes, higher values in root length were noticed under the application of 70 mg/L GA_3_.

**Figure 4:**
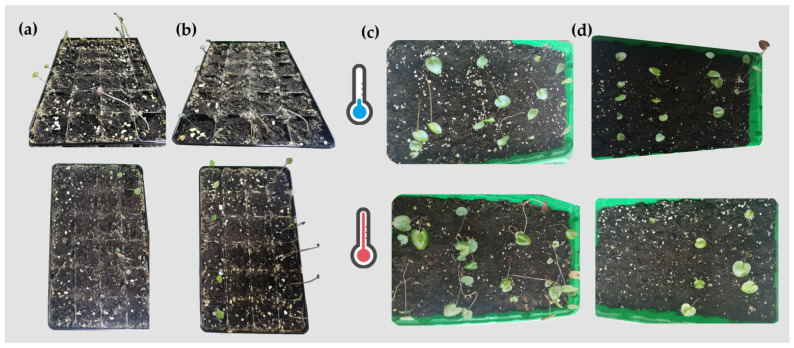
Germination capacity (**a**,**b**) and plant development (**c**,**d**) of heat-resistant C15 (**upper left-a**) and heat-sensitive C2 (**upper right-b**) under ambient temperature or heat stress.

The SVI was significantly different among genotypes and GA_3_ treatments. Under HS, significantly lower SVI values were noticed under control, especially in C7 and C8 with values of 65.25 and 60.26, respectively. With 30 mg/L GA_3,_ higher SVI levels were noticed in C10, followed by C14 and C3. The increasing dose of hormone concentrations led to a higher SVI especially in C9. Following the highest GA_3_ dose (90 mg/L), an increase in SVI was noticed in almost all genotypes except C3 and C7 with values of 59.02 and 422.64, respectively. The highest SVI were noticed in C2 and C13 which had the best development among all genotypes. HS negatively affected the heat-sensitive plant’s development as seen by the relatively low SVI values under control, even with GA_3_ treatment. The highest doses of hormone treatment led to higher SVI in C4 under 70 mg/L, and in C5, C11 and C13 with 90 mg/L GA_3_ treatment. Under AT, the heat-resistant genotypes under control presented relatively low SVI which gradually increased by GA_3_ treatment in a dose-dependent manner (Table 2). Thus, the use of 30 mg/L GA_3_ significantly increased the SVI compared with control, with the highest values in C10, followed by C3. This ascending trend was noticed with the increased dose of GA_3_, which had the strongest effect in C10, C16 and C3 under the 70 mg/L GA_3_, whereas the highest value of SVI with 90 mg/L GA_3_ was noticed in C8 and C10. The heat-sensitive genotypes under control presented significantly different SVI values, with the highest in C5 and C6 and the lowest in C1 and C11, respectively. The use of GA_3_ influenced the plant development in a positive way. Thus, the SVI presented a positive tendency under 30 mg/L GA_3_, which maintained its trend with increased dose of GA_3_ (70 and 90 mg/L). The variation in SVI could be attributed to an enhanced germination rate, which strongly depends on the seedlings’ root and shoot development, along with the stimulation of enzymatic activities (Figure 4).

### 2.3. Hierarchical Clustering and Heat Mapping of Seed Germination and Plant Development Parameters of Heat-Tolerant and Heat-Sensitive Genotypes under Ambient Temperature and Heat Stress Conditions

**Figure 5:**
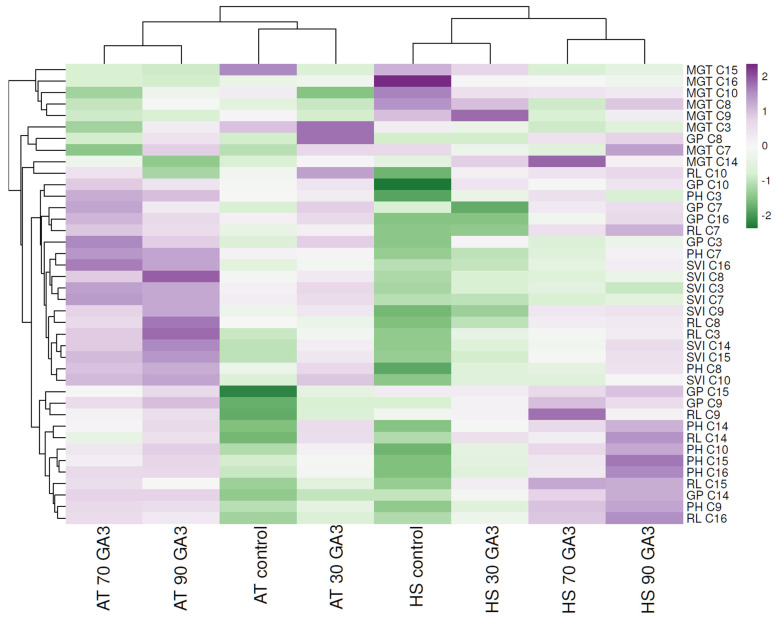
Hierarchical clustering and heatmap visualization of heat-resistant genotypes under ambient temperature and heat stress conditions. Columns indicate the control and GA_3_ treatments, and rows indicate morphological parameters. Cells are colored based on values of seed and plant development, where purple represents a strong positive correlation and green a strongly negative correlation.

Hierarchical clustering and heat mapping were used to visualize similarities and differences between AT, HS and GA_3_ applications. Several heat-resistant genotypes exhibited unusual behavior in HS conditions, namely C3, C7 and C8, as these genotypes showed negative correlations in terms of germination parameters and plant development (plant height) under control compared with GA_3_ application. The other genotypes were found to be less affected by heat stress as compared with the above-mentioned genotypes as shown by the positive correlation between SVI, germination percentage and root length under 70 and 90 mg/L GA_3_. Conversely, a negative correlation was shown in these genotypes under control in the majority of the traits which were drastically influenced by heat stress. Thus, these were referred to as susceptible genotypes as vindicated by their performance trend in Figure 5. As shown in the heatmap, it is clear that GA_3_ treatment elevated the negative effect of HS by inducing seed germination and plant development with increased dose application. In HS under control, a negative correlation in GP, SVI and plant height was noticed as all genotypes were strongly affected by higher temperatures. Following the importance score and the increased GA_3_ application, a positive correlation is shown in terms of plant height and root length, demonstrating the effectiveness of hormone treatment under HS conditions. Except for C3, C7 and C8 which proved to be susceptible to HS even under GA_3_ application, the other genotypes showed tolerance to higher temperatures.

**Figure 6:**
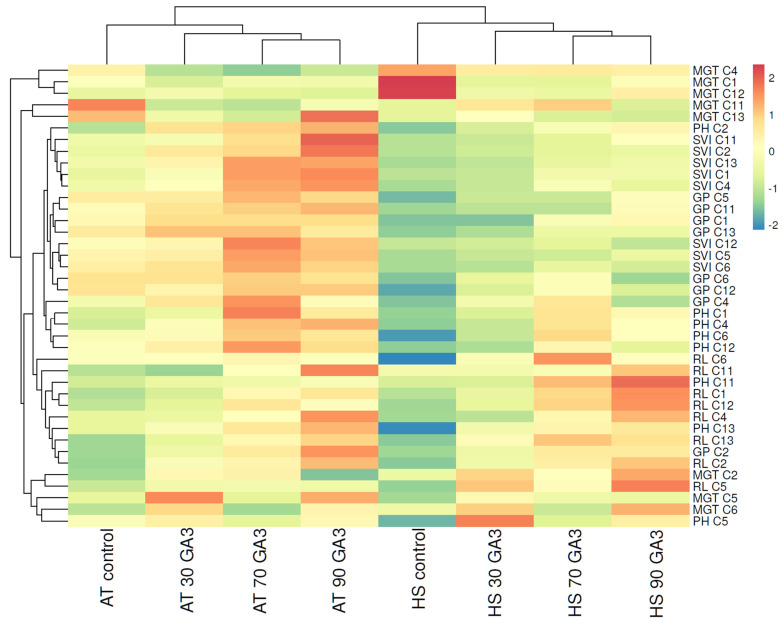
Hierarchical clustering and heatmap visualization of heat-sensitive genotypes under ambient temperature and heat stress conditions. Columns indicate the control and GA_3_ treatments, and rows indicate morphological parameters. Cells are colored based on values of seed and plant development, where red represents a strong positive correlation and blue a strongly negative correlation.

In the case of heat-sensitive genotypes, significant differences were observed under ambient and increased temperatures as observed by the importance scores (Figure 6). Under AT, the genotypes presented increased germination rate and plant development with increased GA_3_ application as shown by their overall positive trend in the red colored gradient strips especially with 70 and 90 mg/L hormone treatment. Under HS, these genotypes showed rather negative values under control conditions, emphasizing their low susceptibility to increased temperatures. Following GA_3_ application, genotypes C4, C6, C11 and C13 possessed higher positive or negative values compared with the other heat-sensitive genotypes. These genotypes were shown to be less affected by heat stress, suggesting their positive response to hormone treatment. As shown in the heatmap, these genotypes presented negative values in terms of germination parameters and positive values in root length compared with the other heat-sensitive genotypes, emphasizing their tolerance to heat stress under GA_3_ application. In the present conditions, both heat-resistant and heat-sensitive genotypes were found to have higher petiole and root lengths and leaf area values as compared with control. Overall, heat-resistant C9, C10, C11, C14 and C16 genotypes are highly resistant to heat stress especially under hormone treatment, whereas the heat-sensitive genotypes, C4, C6, C11 and C13 proved to be less susceptible to HS as illustrated in Figure 6. These genotypes can be further employed in breeding programs by using multiple stress designs and hormone treatments to achieve maximum heat tolerance in crop generations, which may contribute to enhanced productivity.

## 3. Discussion

A prevalent concern due to climate change urges researchers to develop heat-tolerant genotypes to cope with the decline in water sources and rising temperatures so as to diminish their damaging effect on crop development. Utilization of low-cost hormone treatment along with tolerant genotypes is useful in the long term in genetic breeding programs. Moreover, identification of efficient screening techniques to detect heat-resistant plants proves to be significant approach for future studies. At seedling stage, phenotypic screening can be evaluated using destructive methods (fresh and dry weight) and non-destructive ones (image processing, canopy temperature). Moreover, root development was shown to be a reliable predictor of the plant’s response to drought and heat stresses through its direct connection with soil, water relation and nutrient absorption under unfavorable environmental conditions [27]. It was demonstrated that heat stress strongly affects seed germination and vigor causing thermal injury or seed death. Various physiological parameters in plants are also affected by heat stress, resulting mainly in earlier leaf senescence, shoot and root growth inhibition, reduction in flower number and fruit development, all of which eventually lead to loss of crop yield [28,29]. Recently, Siddiqui et al. (2015) demonstrated that growth parameters of faba bean were significantly reduced under heat stress [30]. Seed priming with GA_3_ overcomes seed dormancy mainly due to deterioration of the endosperm layer and activation of embryo development [31]. Pre-sowing treatments with GA_3_ increased seed yield and qualitative characteristics under both drought stress and non-stress conditions compared with genotypes under control (no treatment) [23]. Germination rate of *T. terscheckii* seeds was strongly influenced under light regimes, heat stress and GA_3_ treatments. Increased germination proportion was noticed under ambient temperatures compared with higher temperatures with increased GA_3_ concentrations under both conditions. Conversely, significantly low germination was noticed under darkness only with increased dose of GA_3_ (1000 mg/L) [32]. GA_3_ (60 mg/L) was demonstrated to be a quick and efficient treatment in breaking rice seed dormancy compared with lower concentrations [33]. It was demonstrated that application of GA_3_ between 50 and 100 mg/L proves to be efficient in breaking seed dormancy of *Cyclamen* species under high light exposure treatment. Thus, the highest germination rate in *C. africanum* and *C. hederifolium* was noticed under 50 mg/L GA_3_ treatment, whereas *C. cyprium* presented the highest germination percentage with application of 100 mg/L GA_3_ [4]. Early emergence, high germination percentage and normal seedlings development with least mortality were noticed in seeds of Hevea brasiliensis (rubber) with 100 mg/L GA_3_ treatment [34]. Conversely, the germination rate of Lavandula angustifolia ‘Codreanca’ and ‘Sevtopolis’ were favorably influenced with application of GA_3_ at doses of 200 and 300 mg/L [35]. Industrial hemp (*Cannabis sativa* L.) seed pre-treatments with high levels of GA_3_ (500 and 1000 mg/L) were associated with a decreasing trend in germination, but a positive effect on early growth responses was observed [36]. Priming treatment of *Brassica napus* L. seeds with 300 mg/L GA_3_ showed a significantly increased drought tolerance index compared with control and improved seedling tolerance to drought stress [37]. In the present study, heat stress significantly influenced the germination rate and plant development in heat-tolerant and heat-sensitive genotypes. Both heat-tolerant and heat-sensitive genotypes presented increased germination rate with GA_3_, especially with 70 and 90 mg/L under both AT and HS conditions. Heat-sensitive genotypes positively responded to GA_3_ especially in the case of C1 and C2 which might be selected as resistant genotypes as seen by their high germination rate (73.33–93.33%).

Plant response to increased temperatures depends on several factors in which plant regulators are considered significant and are involved in the mechanisms of susceptibility or tolerance of plants. Under abiotic stress, several proteins (DELLAs) are produced by the induction of different plant hormone levels. Seed priming with optimal concentrations of GA_3_ was proven advantageous to increase early seedling growth under abiotic stress conditions [38]. Under HS, treatment with GA_3_ (288.7 µM) increased the emergence percentage and emergence rate of sweet sorghum (*Sorghum bicolor* L. Moench) under heat stress compared with control. Lower levels of shoot and higher levels in root length and number were noticed under GA_3_ treament under HS [39]. Combined treatment of silicon (Si) and GA_3_ significantly influenced the development of date palm (*Phoenix dactylifera* L.) resulting in alleviation of adverse effect of HS and greatest shoot length (31.87 cm) and root length (11.56 cm) compared with solely hormone treatment and control. By comparison, relatively close results were noticed under sole treatment with GA_3_ emphasizing its usage, alone or in combination with other growth regulators, as an efficient treatment under HS conditions [40]. Exogenous GA_3_ application elevated the adverse effects of HS in Arabidopsis [41]. Heat-tolerant and heat-sensitive perennial ryegrass (*Lolium perenne*) accessions were both affected by HS resulting in decreased plant height and leaf water content, but with delayed negative effect in the tolerant genotype [42]. According to previous reports, the optimal temperature for germination in *Cyclamen* is 15 °C, whereas temperatures above 20 °C lead to inhibition of germination [43]. Supra-optimal temperatures (25–30 °C) inhibit germination with longer exposure (4 weeks) [44]. Moreover, temperatures higher than 25 °C or hypoxia inhibit tuber formation and lead to very elongated tubers which affects the plant’s development [45]. In the present study, heat-resistant genotypes positively responded to GA_3_ treatment in a dose-dependent manner under HS conditions. Elevated levels in SVI were noticed with 90 mg/L GA_3_ with regard to Merengue genotypes, mainly C10, C14 and C15, which presented earlier and higher seedling development under HS conditions. Conversely, heat-sensitive genotypes were negatively affected by HS, whereas GA_3_ acted in a protective way by enhancing seedling development and reducing the adverse effects of higher temperature exposure. Regarding the plant development, increased petiole and plant height were noticed in heat-tolerant genotypes under HS conditions with increased doses of GA_3_, especially with 70 and 90 mg/L treatment regimes.

Regarding the root development under HS conditions, relatively few reports have evaluated their responses with growth hormone treatments. The effect of GA_3_ application on the growth and development of roots was evaluated in paper flower (*Bougainvillea glabra*), jungle geranium (*Ixora coccinea*) and Chinese rose (*Rosa chinensis*) finding the highest levels with the dose of 100 mg/L GA_3_ in all above-mentioned genotypes [46]. In a different study, GA_3_ concentrations between 0.05 and 50 mg/L were applied to several *Pelargonium* (geranium) cultivars to assess their influence on root development. Their results showed that with increased GA_3_ concentration, an increased growth rate and decreased shoot:root ratio were noticed [47]. Exposure of cucumber seedlings to a relatively low root-zone temperature of 16 °C led to significantly lower root growth and development which was reversed by exogenous GA_3_ application [48]. Conversely, an increase in ambient temperature was reported to stimulate GA production, reduce DELLA levels and promote stem elongation in Arabidopsis [49]. Root lengths at seedling stage were severely reduced by HS with significant variations among wheat genotypes. Heat-tolerant genotypes at seedling stage showed less root length decrease compared with heat-susceptible ones [50]. In the present study, significant differences were noticed between root development of heat-sensitive and heat-tolerant genotypes with increased GA_3_ doses. Under HS, heat-resistant genotypes revealed significantly higher root lengths with 70 and 90 mg/L GA_3_ treatment. Conversely, significant differences between AT and HS conditions were noticed only in Petticoat genotypes (C2, and C12) and Metis genotypes (C5 and C6), respectively. Overall, heat-resistant C15 and heat-sensitive C2 might be selected for future studies under different abiotic and/or biotic stresses, according to their highest germination capacity (86.67–100% and 80.0–93.33%, respectively) and plant vigor (116.93–1184.1 and 132.42–509.28, respectively).

## 4. Materials and Methods

### 4.1. Plant Materials

The experiments were carried out at the Advanced Horticultural Research Institute of Transylvania (AHRIT), University of Agricultural Sciences and Veterinary Medicine Cluj-Napoca, Romania, using 16 *Cyclamen* accessions selected for their foliar and ornamental quality. A dual experiment was carried out: plants were maintained under control conditions of optimum temperature and heat stress. The 16 genotypes were divided into two agronomic groups according to their provenance: group 1 (Morel company breeder, Rue de Montourey, Fréjus, France) and group 2 (Schoneveld Breeding, Zeewolde, The Netherlands) (Supplementary Table S1). The plants were sown in May 2021 so that they would reach the juvenile vegetative stage for the heat treatment approximately in June–July 2021.

### 4.2. Growth Regulator Treatment

**Figure 7:**
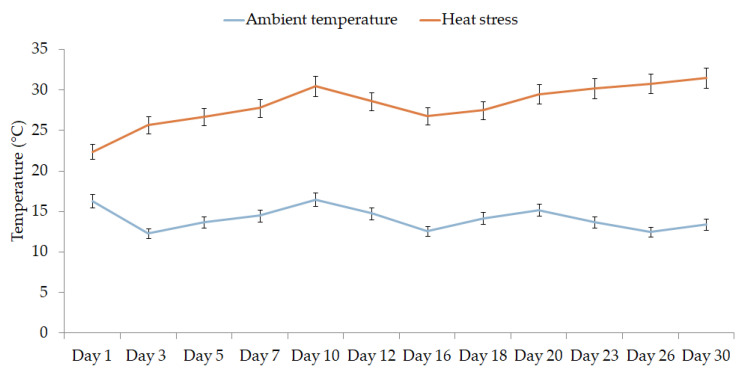
Average of the growth chambers recordings during the experiment.

Seeds of *Cyclamen* genotypes were subjected to hormone seed priming using different concentrations of GA_3_ and using distilled water for control for 24 h according to [38]. Afterward, the seeds were washed three times with distilled water, surface dried and transferred to growth chambers. Seeds were germinated on two-layer filter paper in Petri dishes (14.5 cm diameter) with 10 mL of distilled water and different levels of GA_3_ (0, 30, 70 and 90 mg/L) in altering temperature conditions of either ambient temperature (11–17 °C) or heat stress (23–36 °C) with 60% relative humidity (Figure 7).

### 4.3. Pot Experiment

After germination, the seedlings were transplanted to pots (30 cm length and 14 cm width) and watered according to the plants’ requirements (at 3 days’ interval) with distilled water (control) and GA_3_ solutions in different concentrations. The growing substrate (60/20/20 *v*/*v*) was a mixture of sowing and propagation soil (pH = 6.0) with a content of NPK 0.1:0.01:0.03 m/m%, *Cyclamen* substrate (pH = 6.2) with a content of NPK (1.0:0.1:0.3 m/m%), 70% organic substances and perlite. Seeds germinated in approximately 3 weeks. Afterward, the influence of exposure to ambient temperatures (AT) and heat stress conditions (HS) under the influence of different GA_3_ concentrations on morphological parameters was evaluated. Non-destructive methods were used by scanning all seedlings with the ImageJ Programme 1.52a, Wayne Rasband, National Institutes of Health Bethesda, Maryland, USA for image processing. Plant height, root and petiole lengths (cm), and leaf area were determined for heat-resistant and heat-sensitive genotypes under control and heat stress conditions. The germination percentage [51] was calculated accordingly:

(1) $$\mathrm{GP}=\;\frac{\sum \mathrm{Ns}}{\mathrm{Fs}}\times 100\%$$

where Ns corresponds to the number of seeds at the establishment of the experiment and Fs represents the germinated seeds at the end of the experiment. Germination rate [51,52] was assessed by estimating the mean germination time (MGT) using the following equation:

(2) $$\mathrm{MGT}=\frac{\sum \mathrm{DNs}}{\sum \mathrm{Ns}}$$

where D is the day at the start of the germination test, and Ns is the number of recently germinated seeds on day D [53]. The Seedling Vigor Index (SVI) [52] was evaluated using the equation described below:

(3) $$\mathrm{SVI}\;=\;\mathrm{GP}\;\times \left(\mathrm{Mrl}+\mathrm{Mhl}\right)$$

GP represents the germination percentage, MrL the average root length (mm) and Mhl the average seedling length (mm) [54].

### 4.4. Statistical Analysis

For each randomized block (16 blocks in total), 5 plants per treatment were used (a total of 20 plants per exposure treatment and genotype). The aggregated dataset comprises 16 genotypes of 320 plants (80 control and 240 stress) together with germination parameters and morphological characteristics. Data collected were analyzed with the analysis of variance technique (ANOVA) using HSD Tukey’s test (*p* ≤ 0.05) and presented as the interaction of ambient and high temperature stress and GA_3_ treatment using GraphPad Prism 8.2.1.441. Results were given as average ± standard deviation. Heatmap and dendrograms were generated using the Euclidean distance based on Ward’s algorithm for clustering [55].

## 5. Conclusions

The present study highlights the priming effects with GA_3_ on seed germination and plant development and the most advantageous GA_3_ dosage applied to *Cyclamen* accessions under AT and HS conditions. Moreover, the cultivated germplasm’s genetic diversity for HS tolerance was assessed to select genotypes which possess increased tolerance to heat. Heat-tolerant genotypes, mainly C3 (6.42%), C15 (6.47%) and C16 (5.12%) had the lowest MGT (highest germination rate) with 90 mg/L GA_3_ treatment compared with control. Regarding heat-sensitive genotypes, C11 (5.11%) and C13 (4.28%) which had the lowest MGT values, along with C1 (73.3%) and C2 (80.0%) with a high germination percentage, can be selected as genotypes which might be used in future studies to assess their resistance under heat and/or drought stress, but also subjected to different growth hormone regulators. Under HS in heat-tolerant genotypes with 70 mg/L GA_3,_ the genotypes that presented the highest plant development were C9, C10 and C14 with values between 12.79 and 15.43 cm. The above genotypes which presented the highest values in germination and plant development might be selected as heat-resistant genotypes to be deposited in germplasm banks and used in further amelioration programs under biotic and/or abiotic stresses to develop resistant genotypes.

## Figures and Tables

**Table 1:** Effects of GA_3_ concentrations (mg/L) on germination parameters of the selected heat-resistant and heat-sensitive *Cyclamen* genotypes under ambient temperatures and heat stress.

Parameters	Genotype	GA_3_ Concentrations Treatment under AT				GA_3_ Concentrations Treatment under HS			
		0 mg/L GA_3_	30 mg/L GA_3_	70 mg/L GA_3_	90 mg/L GA_3_	0 mg/L	30 mg/L	70 mg/L	90 mg/L GA_3_
						GA_3_	GA_3_	GA_3_	
**Germination percentage (%)**	**Heat-resistant genotypes**								
	C3	80.0 ± 8.0 ^b^	93.3 ± 9.1 ^a^	100.0 ± 10.0 ^a^	93.3 ± 9.4 ^a^	73.4 ± 3.3 ^c^	86.7 ± 2.1 ^b^	80.0 ± 2.0 ^c^	83.3 ± 3.4 ^c^
	C7	73.3 ± 9.1 ^b^	93.3 ± 9.5 ^ab^	100.0 ± 10.2 ^a^	86.7 ± 8.8 ^ab^	73.3 ± 5.1 ^c^	60.0 ± 1.8 ^d^	86.7 ± 3.1 ^de^	90.0 ± 1.8 ^b^
	C8	86.7 ± 8.3 ^a^	100.0 ± 8.9 ^a^	86.7 ± 8.0 ^a^	93.3 ± 8.8 ^a^	86.7 ± 4.5 ^a^	86.7 ± 5.7 ^b^	93.3 ± 6.0 ^ab^	95.0 ± 3.4 ^ab^
	C9	60.0 ± 8.0 ^b^	73.3 ± 8.8 ^ab^	93.3 ± 8.3 ^ab^	100.0 ± 9.7 ^a^	73.3 ± 4.6 ^c^	86.7 ± 2.9 ^b^	100.0 ± 3.3 ^a^	93.3 ± 1.7 ^b^
	C10	86.7 ± 7.9 ^b^	93.3 ± 9.0 ^ab^	100.0 ± 8.9 ^a^	93.3 ± 9.5 ^ab^	53.4 ± 6.7 ^d^	93.3 ± 6.6 ^a^	86.7 ± 2.8 ^d^	93.3 ± 2.5 ^b^
	C14	53.3 ± 5.3 ^c^	60.0 ± 6.0 ^b^	86.7 ± 7.8 ^a^	86.7 ± 7.8 ^a^	60.0 ± 5.5 ^d^	73.3 ± 2.0 ^c^	86.7 ± 5.8 ^de^	93.3 ± 3.3 ^ab^
	C15	40.0 ± 4.1 ^c^	73.3 ± 7.3 ^b^	80.0 ± 7.9 ^b^	93.3 ± 9.2 ^a^	86.7 ± 4.5 ^a^	86.7 ± 6.0 ^ab^	93.3 ± 5.2 ^ab^	100.0 ± 4.0 ^a^
	C16	90.0 ± 8.4 ^b^	95.0 ± 9.5 ^ab^	100.0 ± 9.7 ^a^	95.0 ± 9.1 ^ab^	73.3 ± 4.4 ^c^	73.3 ± 3.5 ^c^	86.7 ± 2.7 ^d^	95.0 ± 1.1 ^b^
	**Heat-sensitive genotypes**								
	C1	80.0 ± 7.9 ^a^	93.3 ± 8.6 ^a^	93.3 ± 8.8 ^a^	93.3 ± 9.4 ^a^	40.0 ± 3.8 ^e^	40.0 ± 2.5 ^f^	73.3 ± 0.8 ^f^	80.0 ± 2.4 ^c^
	C2	80.0 ± 7.6 ^b^	86.7 ± 8.8 ^ab^	93.3 ± 9.3 ^a^	100.0 ± 9.5 ^a^	80.0 ± 0.6 ^b^	86.7 ± 2.6 ^b^	93.3 ± 1.5 ^b^	93.3 ± 2.1 ^b^
	C4	73.3 ± 7.4 ^b^	86.7 ± 8.3 ^b^	100.0 ± 9.6 ^a^	80.0 ± 7.8 ^b^	53.4 ± 2.4 ^d^	73.3 ± 5.3 ^c^	86.7 ± 1.6 ^d^	60.0 ± 2.8 ^e^
	C5	80.0 ± 7.6 ^b^	80.0 ± 7.3 ^b^	93.3 ± 9.7 ^a^	86.7 ± 8.9 ^ab^	40.0 ± 5.6 ^e^	53.3 ± 2.3 ^e^	53.3 ± 1.9 ^h^	73.3 ± 3.9 ^d^
	C6	93.3 ± 9.0 ^a^	93.3 ± 8.7 ^a^	100.0 ± 9.9 ^a^	93.3 ± 8.7 ^a^	33.3 ± 3.2 ^ef^	60.0 ± 3.3 ^d^	73.3 ± 2.9 ^f^	40.0 ± 1.7 ^g^
	C11	73.3 ± 6.8 ^b^	86.7 ± 8.0 ^ab^	93.3 ± 8.9 ^a^	100.0 ± 9.9 ^a^	33.3 ± 5.8 ^ef^	40.0 ± 3.3 ^f^	40.0 ± 1.6 ^i^	73.3 ± 7.1 ^d^
	C12	86.7 ± 8.2 ^a^	80.0 ± 7.9 ^a^	93.3 ± 8.9 ^a^	93.3 ± 9.0 ^a^	40.0 ± 7.7 ^e^	60.0 ± 4.9 ^d^	73.3 ± 3.1 ^f^	60.0 ± 8.0 ^ef^
	C13	86.7 ± 9.2 ^b^	100.0 ± 9.9 ^a^	100.0 ± 10.4 ^a^	86.7 ± 8.4 ^b^	33.3 ± 7.6 ^f^	40.0 ± 5.6 ^f^	60.0 ± 3.4 ^g^	60.0 ± 5.6 ^ef^
**MGT**	**Heat-resistant genotypes**								
	C3	13.90 ± 5.7 ^a^	17.64 ± 7.2 ^a^	3.24 ± 2.0 ^b^	10.49 ± 6.0 ^a^	10.31 ± 3.49 ^e^	7.50 ± 5.40 ^g^	5.088 ± 2.34 ^ef^	6.42 ± 3.84 ^e^
	C7	4.02 ± 2.1 ^ab^	8.70 ± 6.1 ^a^	3.03 ± 1.5 ^b^	8.98 ± 5.9 ^a^	8.48 ± 0.91 ^f^	6.13 ± 3.06 ^gh^	5.35 ± 2.33 ^e^	10.34 ± 6.56 ^d^
	C8	6.18 ± 3.3 ^a^	4.25 ± 2.2 ^a^	3.90 ± 2.0 ^a^	8.66 ± 7.7 ^a^	16.91 ± 2.66 ^d^	14.33 ± 2.86 ^cd^	4.62 ± 2.08 ^f^	13.85 ± 1.87 ^b^
	C9	11.44 ± 5.0 ^a^	3.71 ± 2.0 ^b^	3.39 ± 1.9 ^b^	4.32 ± 2.2 ^b^	19.35 ± 5.48 ^c^	26.24 ± 1.90 ^a^	4.68 ± 2.49 ^f^	12.98 ± 3.71 ^c^
	C10	11.57 ± 6.1 ^a^	2.34 ± 0.9 ^b^	3.70 ± 2.0 ^ab^	9.00 ± 7.9 ^a^	18.72 ± 4.46 ^cd^	12.86 ± 7.89 ^de^	12.28 ± 1.69 ^c^	11.89 ± 3.94 ^d^
	C14	7.01 ± 3.1 ^a^	12.19 ± 7.3 ^a^	9.89 ± 6.2 ^a^	2.65 ± 0.3 ^b^	8.42 ± 3.53 ^f^	16.77 ± 3.52 ^b^	23.51 ± 3.16 ^a^	12.69 ± 2.90 ^c^
	C15	19.95 ± 6.2 ^a^	4.82 ±1.9 ^b^	4.25 ± 1.6 ^b^	3.72 ± 1.6 ^b^	17.10 ± 2.08 ^d^	14.30 ± 1.09 ^cd^	4.54 ± 1.99 ^g^	6.47 ± 2.66 ^e^
	C16	4.26 ± 2.1 ^a^	5.14 ± 2.6 ^a^	2.01 ± 0.7 ^b^	1.76 ± 0.6 ^b^	19.39 ± 3.95 ^c^	6.47 ± 3.00 ^gh^	6.05 ± 2.64 ^e^	5.12 ± 2.14 ^f^
	**Heat-sensitive genotypes**								
	C1	13.43 ± 7.4 ^a^	2.49 ± 0.7 ^a^	10.10 ± 6.0 ^a^	9.06 ± 4.8 ^a^	44.89 ± 4.94 ^a^	5.21 ± 1.81 ^h^	4.31 ± 3.01 ^g^	12.98 ± 5.87 ^c^
	C2	4.77 ± 2.1 ^a^	10.43 ± 4.3 ^a^	10.91 ± 4.6 ^a^	3.74 ± 1.6 ^a^	7.98 ± 4.88 ^g^	13.069 ± 3.81 ^de^	9.944 ± 3.30 ^d^	14.61 ± 2.48 ^a^
	C4	11.55 ± 6.4 ^a^	3.69 ± 1.5 ^b^	1.64 ± 0.4 ^c^	4.54 ± 1.7 ^b^	17.05 ± 2.62 ^d^	12.33 ± 2.67 ^e^	12.63 ± 4.72 ^c^	11.80 ± 1.70 ^d^
	C5	10.37 ± 4.8 ^ab^	22.73 ± 12.5 ^a^	9.63 ± 4.4 ^b^	21.09 ± 12.7 ^a^	6.01 ± 3.46 ^h^	14.95 ± 1.87 ^c^	11.35 ± 3.74 ^d^	10.82 ± 3.57 ^d^
	C6	3.86 ± 2.3 ^ab^	11.01 ± 8.0 ^a^	2.90 ± 1.9 ^b^	9.01 ± 5.7 ^a^	6.27 ± 3.02 ^h^	11.34 ± 4.68 ^e^	4.33 ± 2.19 ^g^	12.46 ± 1.67 ^c^
	C11	20.54 ± 12.1 ^a^	3.94 ± 1.6 ^b^	3.18 ± 0.6 ^b^	8.78 ± 3.1 ^b^	6.08 ± 3.18 ^h^	14.02 ± 3.11 ^d^	16.23 ± 1.13 ^b^	5.11 ± 2.13 ^f^
	C12	4.61 ± 1.6 ^a^	7.27 ± 3.1 ^a^	3.36 ± 1.5 ^ab^	3.18 ± 1.5 ^b^	32.35 ± 1.11 ^b^	5.88 ± 3.19 ^h^	5.32 ± 2.49 ^e^	14.45 ± 6.95 ^a^
	C13	17.87 ± 9.1 ^a^	7.60 ± 3.6 ^b^	4.18 ± 3.4 ^a^	22.78 ± 13.8 ^a^	5.84 ± 3.19 ^h^	10.60 ± 4.98 ^ef^	4.95 ± 2.49 ^ef^	4.28 ± 1.96 ^f^

Values represent the mean ± standard deviations of three independent determinations. Different letters (a,b,c,d,e,f,g,h,i) within a column denote significant differences (*p* < 0.05).

**Table 2:** Effects of GA_3_ concentrations (mg/L) on plant development of the selected heat-resistant and heat-sensitive *Cyclamen* genotypes under ambient temperatures and heat stress.

Parameters	Genotype	GA_3_ Concentrations Treatment under AT				GA_3_ Concentrations Treatment under HS			
		0 mg/L GA_3_	30 mg/L GA_3_	70 mg/L GA_3_	90 mg/L GA_3_	0 mg/L GA_3_	30 mg/L GA_3_	70 mg/L GA_3_	90 mg/L GA_3_
	**Heat-resistant genotypes**								
**Plant height (cm)**	C3	7.39 ± 0.2 ^b^	8.10 ± 0.5 ^ab^	10.74 ± 1.1 ^a^	10.17 ± 0.5 ^a^	2.31 ± 0.0 ^ef^	6.46 ± 1.1 ^cd^	8.78 ± 1.2 ^d^	5.41 ± 0.2 ^f^
	C7	7.04 ± 0.7 ^c^	6.83 ± 0.9 ^c^	12.11 ± 0.9 ^a^	11.44 + 0.7 ^b^	1.64 ± 0.1 ^g^	2.85 ± 0.5 ^h^	4.41 ± 0.1 ^g^	7.01 + 0.3 ^de^
	C8	7.39 ± 0.4 ^b^	11.15 ± 1.0 ^a^	12.12 ± 0.7 ^a^	12.99 + 1.1 ^a^	1.83 ± 0.0 ^f^	6.17 ± 0.9 ^de^	6.89 ± 0.2 ^e^	10.22 + 1.2 ^c^
	C9	5.24 ± 0.1 ^c^	7.75 ± 0.6 ^b^	12.15 ± 0.8 ^a^	11.82 + 0.1 ^a^	3.94 ± 0.3 ^c^	7.04 ± 0.4 ^c^	13.54 ± 1.7 ^ab^	14.89 + 0.4 ^b^
	C10	7.93 ± 0.2 ^c^	13.25 ± 0.5 ^b^	14.06 ± 0.2 ^b^	16.07 ± 0.6 ^a^	5.52 ± 0.0 ^b^	10.77 ± 0.7 ^a^	15.43 ± 1.1 ^a^	17.98 ± 0.7 ^a^
	C14	6.08 ± 0.8 ^c^	12.63 ± 0.1 ^b^	11.02 ± 0.4 ^d^	12.76 ± 0.3 ^a^	6.27 ± 0.4 ^a^	10.59 ± 0.4 ^a^	12.79 ± 0.4 ^b^	14.29 ± 0.8 ^b^
	C15	5.74 ± 0.3 ^d^	8.88 ± 0.5 ^c^	9.94 ± 0.7 ^b^	12.07 ± 1.2 ^a^	2.94 ± 0.2 ^d^	7.14 ± 0.6 ^c^	10.55 ± 1.5 ^c^	16.17 ± 1.8 ^a^
	C16	5.72 ± 0.5 ^c^	7.82 ± 0.3 ^b^	9.94 ± 0.2 ^a^	10.06 ± 1.2 ^a^	4.12 ± 0.1 ^c^	6.64 ± 0.3 ^cd^	8.96 ± 0.1 ^d^	12.35 ± 1.1 ^bc^
	**Heat-sensitive genotypes**								
	C1	3.24 ± 0.4 ^d^	3.98 ± 0.8 ^c^	9.76 ± 1.5 ^a^	6.73 ± 0.8 ^b^	1.56 ± 0.2 ^g^	3.29 ± 0.3 ^fg^	7.19 ± 1.1 ^e^	5.90 ± 0.7 ^f^
	C2	2.51 ± 0.1 ^c^	7.92 ± 0.4 ^b^	8.47 ± 0.8 ^a^	9.43 ± 1.0 ^a^	1.31 ± 0.3 ^h^	3.45 ± 0.4 ^fg^	5.43 ± 0.8 ^f^	6.60 ± 3.5 ^e^
	C4	4.10 ± 0.3 ^c^	6.70 ± 2.1 ^b^	10.08 ± 0.9 ^a^	10.50 + 1.1 ^a^	2.27 ± 0.2 ^f^	3.81 ± 0.8 ^f^	8.85 ± 0.6 ^d^	7.10 + 0.7 ^de^
	C5	6.96 ± 0.2 ^c^	7.18 ± 0.3 ^a^	6.72 ± 0.4 ^d^	7.09 + 0.5 ^b^	6.24 ± 0.4 ^a^	7.72 ± 0.3 ^b^	6.68 ± 0.5 ^e^	7.19 + 0.5 ^d^
	C6	5.72 ± 0.4 ^d^	6.55 ± 0.3 ^c^	8.47 ± 0.9 ^a^	7.58 + 0.6 ^b^	1.23 ± 0.2 ^h^	3.62 ± 0.3 ^f^	8.22 ± 0.3 ^d^	6.24 + 2.4 ^e^
	C11	5.60 ± 0.2 ^a^	6.31 ± 0.7 ^a^	6.54 ± 0.4 ^a^	7.02 ± 0.8 ^a^	5.74 ± 0.2 ^b^	5.76 ± 0.2 ^e^	9.44 ± 0.9 ^c^	10.80 ± 0.3 ^c^
	C12	5.73 ± 0.4 ^c^	6.60 ± 0.5 ^b^	8.89 ± 1.0 ^a^	7.50 ± 0.7 ^a^	2.56 ± 0.1 ^d^	3.19 ± 0.3 ^gh^	6.37 ± 1.1 ^ef^	4.44 ± 0.2 ^g^
	C13	5.59 ± 0.6 ^d^	6.75 ± 0.5 ^c^	8.24 ± 0.4 ^b^	9.51 ± 0.8 ^a^	2.22 ± 0.3 ^f^	6.19 ± 0.5 ^d^	7.59 ± 0.8 ^de^	8.19 ± 0.9 ^d^
**Seedling Vigor Index (SVI**)	**Heat-resistant genotypes**								
	C3	1095.9 ± 1.4 ^d^	1476.2 ± 1.3 ^c^	1936.5 ± 1.2 ^a^	1844.4 ± 0.9 ^b^	167.17 ± 9.8 ^e^	489.88 ± 10.1 ^c^	647.3 ± 15.7 ^cd^	359.02 ± 8.4 ^h^
	C7	965.3 ± 2.3 ^d^	1228.2 ± 0.9 ^c^	1795.5 ± 2.1 ^a^	1683.7 ± 3.1 ^b^	65.25 ± 9.8 ^hi^	78.66 ± 5.5 ^i^	249.26 ± 8.7 ^j^	422.64 ± 10.3 ^g^
	C8	797.7 ± 1.2 ^d^	1102.3 ± 3.1 ^c^	1499.8 ± 0.9 ^b^	2229.2 ± 1.6 ^a^	60.26 ± 9.9 ^i^	342.16 ± 15.6 ^e^	432.91 ± 6.5 ^f^	673.11 ± 9.8 ^d^
	C9	849.6 ± 1.3 ^d^	1133.1 ± 1.8 ^c^	1331.4 ± 0.9 ^b^	1562.1 ± 1.2 ^a^	215.45 ± 11.9 ^d^	375.10 ± 11.0 ^e^	1136.54 ± 17.8 ^a^	1190.01 ± 13.8 ^b^
	C10	803.4 ± 1.3 ^d^	1916.0 ± 0.9 ^c^	1997.4 ± 1.5 ^b^	2168.3 ± 2.6 ^a^	263.29 ± 12.1 ^c^	856.54 ± 16.7 ^a^	858.50 ± 9.12 ^b^	1295.57 ± 7.8 ^a^
	C14	504.7 ± 1.2 ^d^	1044.8 ± 1.0 ^c^	1326.2 ± 0.5 ^b^	1635.0 ± 0.3 ^a^	335.21 ± 12.4 ^a^	671.95 ± 12.1 ^b^	867.20 ± 10.9 ^b^	1216.39 ± 15.9 ^a^
	C15	317.3 ± 0.3 ^d^	1097.1 ± 0.5 ^c^	1505.7 ± 0.7 ^b^	1727.8 ± 1.2 ^a^	116.93 ± 3.6 ^f^	409.38 ± 13.0 ^d^	844.30 ± 9.7 ^b^	1184.10 ± 20.7 ^b^
	C16	601.0 ± 0.5 ^d^	883.7 ± 0.7 ^ac^	1976.1 ± 0.0 ^a^	1763.0 ± 1.2 ^b^	303.88 ± 7.9 ^b^	361.43 ± 10.0 ^e^	676.57 ± 6.3 ^c^	1068.72 ± 23.4 ^c^
	**Heat-sensitive genotypes**								
	C1	333.8 ± 1.7 ^d^	563.3 ± 3.4 ^c^	1261.5 ± 5.3 ^b^	1354.7 ± 0.3 ^a^	79.4 ± 8.3 ^h^	113.9 ± 3.3 ^h^	439.5 ± 14.5 ^f^	449.1 ± 7.8 ^fg^
	C2	501.2 ± 0.4 ^d^	1146.0 ± 0.8 ^c^	1306.4 ± 1.2 ^b^	1829.0 ± 2.5 ^a^	132.42 ± 11.1 ^f^	258.1 ± 14.5 ^f^	408.24 ± 14.2 ^g^	509.28 ± 8.5 ^f^
	C4	492.8 ± 1.5 ^d^	687.5 ± 0.9 ^c^	1245.6 ± 0.9 ^b^	1319.5 ± 2.5 ^a^	67.496 ± 13.4 ^hi^	198.46 ± 5.6 ^g^	591.72 ± 20.9 ^d^	371.26 ± 9.9 ^h^
	C5	976.1 ± 1.3 ^d^	1041.7 ± 0.9 ^c^	1524.1 ± 0.8 ^a^	1358.4 ± 1.3 ^b^	146.22 ± 10.9 ^ef^	274.20 ± 7.6 ^f^	327.88 ± 12.4 ^h^	533.99 ± 5.6 ^e^
	C6	1108.6 ± 2.4 ^d^	1240.3 ± 3.1 ^c^	1657.5 ± 0.8 ^a^	1401.9 ± 1.2 ^b^	16.71 ± 4.6 ^j^	84.61 ± 6.9 ^i^	480.35 ± 10.9 ^e^	249.41 ± 9.7 ^i^
	C11	385.9 ± 2.4 ^d^	466.5 ± 2.1 ^c^	830.3 ± 3.1 ^b^	1276.2 ± 0.7 ^a^	107.30 ± 9.9 ^g^	173.79 ± 14.5 ^gh^	279.35 ± 11.3 ^i^	565.46 ± 5.6 ^e^
	C12	746.0 ± 0.9 ^d^	830.2 ± 2.1 ^c^	1614.7 ± 3.1 ^a^	1284.2 ± 2.6 ^b^	94.12 ± 12.7 ^g^	160.85 ± 18.8 ^gh^	304.41 ± 12.8 ^h^	71.94 ± 6.2 ^j^
	C13	543.5 ± 0.6 ^d^	855.2 ± 2.3 ^c^	1379.1 ± 0.4 ^a^	1358.0 ± 0.8 ^b^	74.90 ± 13.5 ^h^	200.03 ± 11.8 ^g^	440.02 ± 13.7 ^f^	534.29 ± 8.8 ^e^

Values represent the mean ± standard deviations of three independent determinations. Different letters (ab,c,d,e,f,g,h,i,j) within a column denote significant differences (*p* < 0.05).

## Data Availability

Not applicable.

## Supplementary Materials

The following supporting information can be downloaded at: https://www.mdpi.com/article/10.3390/plants11141868/s1, Table S1: Overview of 16 *Cyclamen* genotypes used in this study.
