# Supplementary material for: Exogenously Applied GA3 Enhances Morphological Parameters of Tolerant and Sensitive Cyclamen persicum Genotypes under Ambient Temperature and Heat Stress Conditions
Source: Plants (Basel). 2022 Jul 18;11(14):1868. doi: 10.3390/plants11141868 (PMC9316198; doi:10.3390/plants11141868)
Supplement: Supplementary file 1 [file plants-11-01868-s001.zip › plants-1814018-supplementary.pdf]

Table S1. Overview of 16 Cyclamen genotypes used in this study

Species	Sample ID	Variety	Agronomic group	Geographical origin	Resistance
Cyclamen persicum Mill. genotypes	C1	Petticoat pure white	1	The Netherlands	Cold
	C2	Petticoat light eye	1	The Netherlands	Cold
	C3	Smartiz Victoria	2	France	Moderate heat
	C4	Smartiz Violet Fonce	1	The Netherlands	Low heat
	C5	Metis Victoria	1	The Netherlands	Cold
	C6	Metis Blank pur	1	The Netherlands	Cold
	C7	Halios Falbala	1	The Netherlands	Moderate heat
	C8	Latinia Pipoca	1	The Netherlands	Botrytis and moderate heat
	C9	Jive Salmon Red	2	France	Moderate heat
	C10	Merengue Salmon red	2	France	Heat
	C11	Verano st.	1	The Netherlands	Cold
	C12	Petticoat dark violet	2	France	Cold
	C13	Metis Origami	1	The Netherlands	Cold
	C14	Merengue Pure white	2	France	Heat
	C15	Merengue Magenta	2	France	Heat
	C16	Metis Silver leaf	1	The Netherlands	Heat
